# Water Supply of London

**Published:** 1857-01

**Authors:** 


### THE WATER SUPPLY OF LONDON.

LONDON is now supplied with water by nine companies; five of these derive the water from the Thames; the others have special sources of supply. The first five in the subjoined list use Thames water.

*Comparison of the Average Results obtained in the Analysis of Companies' Waters in* 1856, *with those of the Analysis made in* 1851.

| Description of Water. | Date of Taking. | Hardness. | | | Solid Constituents, Grains per Gallon. | | |
|---|---|---|---|---|---|---|---|
| | | Total. | Permanent. | Temporary. | Total solid Residue. | Organic Matter. | Inorg. Matter. |
| Grand Junction ...... | 1851 | 14·00 | — | — | 21·72 | 3·07 | 18.65 |
| | 1856 | 14·87 | 7·92 | 6·95 | 22·59 | 1·38 | 21·21 |
| West Middlesex ...... | 1851 | 14·60 | — | — | 22·67 | 2·75 | 19·92 |
| | 1856 | 14·28 | 8·12 | 6·16 | 21·03 | 0·96 | 20·07 |
| Chelsea ............ | 1851 | 14·44 | — | — | 21·28 | 2·38 | 18·90 |
| | 1856 | 13·80 | 8·63 | 5·17 | 22·79 | 1·42 | 21·37 |
| Southwark & Vauxhall | 1851 | 15·00 | — | — | 21·08 | 1·51 | 19·57 |
| | 1856 | 13·59 | 8·22 | 5·37 | 21·19 | 1·37 | 19·82 |
| Lambeth ............ | 1851 | 14·16 | — | — | 20·40 | 2·59 | 17·81 |
| | 1856 | 11·98 | 7·82 | 4·16 | 19·84 | 1·33 | 18·51 |
| New River .......... | 1851 | 14·9 | — | — | 19·50 | 2·79 | 16·71 |
| | 1856 | 13·4 | 7·8 | 5·6 | 21·78 | 0·968 | 20·812 |
| East London ........ | 1851 | 15·00 | — | — | 23·51 | 4·12 | 19·39 |
| | 1856 | 13·98 | 7·53 | 6·45 | 22·05 | 1·09 | 20·96 |
| Kent................ | 1851 | 16·00 | — | — | 29·71 | 2·61 | 27·10 |
| | 1856 | 12·03 | 10·1 | 1·93 | 26·10 | 1·37 | 24·73 |
| Hampstead .......... | 1851 | 9·8 | — | — | 35·41 | 1·84 | 33·57 |
| | 1856 | 7·43 | 7·41 | 0·02 | 29·19 | 1·45 | 27·74 |

This table shows that the hardness of the waters in 1856 was, with one exception, somewhat less than in 1851; the diminution, however, with one or two exceptions, is only trifling. The total amount of solid matter in the two years likewise exhibits but unimportant fluctuations.

*A very considerable diminution, however, is observed in the amount of organic matter.*

In fact, in 1856, the waters supplied to the metropolis contained not more than one-half of the organic matter which was present in the year 1851.

From several samples of water taken for analyses above the sources of the London supply, it was found that there was a very susceptible increase of matters in suspension in the water in its progress downwards from one point to the other, and this increase was especially noticeable after passing Windsor and Eton, two towns of which the drainage has been lately carried out to great extent, without the adoption of any means for avoiding pollution of the river.

Dr. Clark's process for softening water that is rendered hard by carbonate of lime, by the addition of more lime so as to precipitate an insoluble salt, need be considered no longer as a beautiful chemical theory, but as a plan practically carried out now for two years past at the Plumstead, Woolwich, and Charlton Waterworks, with eminent advantage and success.

The softening process is applicable with most advantage, not to river waters but to clear spring waters, such as are usually derived from wells in the chalk, or to other waters of which the hardness is derived from carbonate of lime, but which have acquired no tinge or discolouring admixture.

In this case the precipitated pure carbonate of lime is stated to possess considerable commercial value as an excellent material for whiting, which, according to Mr. Homersham's calculations, would nearly pay for the whole working expenses, and interest on the capital expended for establishing the process. The public would, in such case, enjoy all the advantages of the system almost free of cost.

If the process were applied to a water which has taken up any impurity or discolouring matter, as the water of the Thames, for instance, it is admitted that the precipitate would be valueless ; but, notwithstanding this, looking at all the comfort and advantages to be derived from the use of a soft and pure water, we believe that there are few cases in which we should hesitate to recommend its adoption.—(From a Report to the Board of Health on Metropolis Water Supply. By Professor Hofmann and Mr. Blyth. 1856.)

